# Supplementary material for: From “one big clumsy mess” to “a fundamental part of my character.” Autistic adults’ experiences of motor coordination
Source: PLoS One. 2023 Jun 2;18(6):e0286753. doi: 10.1371/journal.pone.0286753 (PMC10237488; doi:10.1371/journal.pone.0286753)
Supplement: S1 File — (DOCX) [file pone.0286753.s002.docx]

**S2: Focus Group Interview Schedule**

Intro: Hi, I am [name, student on the MRes Psychology degree/Co-director of the Body, Eye and Movement lab].

For Zoom – check everyone can hear and we can hear them

For Skype – check everyone has posted a comment to say they are here

Thank you all for being here today for this focus group. Our research aims to understand the motor coordination difficulties autistic people experience and to do that we would love to hear about your experiences and opinions. It is important to know that there are no right or wrong answers. Everyone’s input is equally important. We are interested in listening to what you have to say. I hope everyone has read through the brief and understood everything on there. Is there anyone that would like to ask any questions about that before we begin? [wait for questions]. If anyone feels distressed or wants to leave at any point, you are free to do so however, we hope you can all stay to give us an insight into your experiences. This focus group (audio only) will be recorded or the text chat saved but everything said today will be kept confidential and used for research purposes only. It is important everyone respects each other’s opinions and privacy outside of this focus group. If that is okay with everyone, let’s make a start!

For Skype text chat: Revisit guidelines and check everyone knows how to post emojis, quote previous text and send private message to researcher

**Definitions and descriptions**

1. [Starting with checking definitions] How do you refer to motor coordination difficulties? What type of difficulties do you think of when referring to [insert chose definition]?
   1. What does gross motor skills mean for you?
   2. What does fine motor skills mean to you?

[at this point check that the group is referring to all the aspects that cover motor coordination difficulties. Indicate will use the term “motor coordination difficulties” to cover all their definitions, but they are free to continue to use their preferred definition]

1. What kinds of motor coordination difficulties do you experience?
   1. How is your coordination for tasks involving eye-hand coordination (manual tasks, fingers, upper body), tasks that involve balance, full body coordination or walking (include asking about trips and falls)?
   2. Are there motor coordination difficulties that are more apparent than others (e.g. fine motor more that gross)?
   3. Are there any aspects of coordination that you find less difficult or are good at?
2. How do you feel your motor difficulties affect your daily experience?
   1. Daily living skills
   2. Social aspects
   3. Work
   4. Hobbies (Sports, musical instruments, dance)
   5. Have your motor difficulties ever excluded you from participating sports and/or social activities? If so, how, and why…
   6. Do other people comment on your coordination skills?
   7. How do your motor difficulties make you feel?
   8. Have you ever felt differently about yourself due to your motor difficulties?
3. Are your coordination difficulties affected by anything
   1. Environments (clutter, objects)
   2. Preferences for certain tools
   3. Stress
4. How well do you learn new movement tasks?
   1. Have you created any strategies to adapt to your motor difficulties in order to complete tasks? Do you think it takes your longer to learn new motor tasks than other people?
   2. How well do you adapt to a new environment (e.g. cooking in a different kitchen) or using a different version of the same tool?
   3. Is there anything that helps with the motor difficulties you experience?
   4. Do you imagine yourself performing the action - does this help? Do you, or have you ever run through a movement in your head before conducting it (e.g. imagining picking a mug up before you actually do)?
   5. Have you ever developed any strategies to hide your motor difficulties?
5. How have your motor coordination difficulties improved/worsened over time?
   1. Have they remained the same from childhood to adulthood (and older age)
      1. Have you experienced differences in motor abilities in your lifetime? Have you experienced changes in your motor abilities at different ages?
   2. What do you think are the reasons for the changes? How have you adapted?
6. For those of you who have other diagnoses, do you think these motor coordination difficulties are associated with one, or more than one of your diagnoses?
   1. Do you think there are particular coordination difficulties that appear autism specific/more common in autistic people?

Prompts:

Could you talk a little more about that?

So what do you think about this? [directed towards someone with an opposing opinion or someone who hasn’t talked much]

Do other people have an opinion on this?
