# Supplementary material for: From “one big clumsy mess” to “a fundamental part of my character.” Autistic adults’ experiences of motor coordination
Source: PLoS One. 2023 Jun 2;18(6):e0286753. doi: 10.1371/journal.pone.0286753 (PMC10237488; doi:10.1371/journal.pone.0286753)
Supplement: S1 Table — (DOCX) [file pone.0286753.s003.docx]

**Table S3 detailing the different semantic codes that were used to build the themes**

| **Theme 1: Motor coordination difficulties are pervasive and variable**  ***Subtheme: Nature of motor issues***   - Classification and terminology used for motor coordination difficulties - Nature of motor coordination issues - Effect of motor coordination difficulties on activities - Other people notice the motor coordination difficulties - Changes in coordination ability over time   ***Subtheme: Coordination can have a spiky profile***   - Spiky profile - Aspects of motor tasks are performed well/motor coordination abilities   ***Subtheme: Variable awareness and acceptance of motor coordination difficulties***   - Understanding of motor coordination issues - Effect of diagnosis - Relationship with dyspraxia - Awareness of motor coordination issues - Integration of difficulties into self |
| --- |
| **Theme 2: Motor coordination is an active process**   - Coordination is an active process and requires concentration - Coordination requires effort and is fatiguing - Multiple demands on processing affect coordination - Movements need to be planned rather than reactive and fast - Impact of sensory on motor - Influences on coordination |
| **Theme 3: Motor coordination difficulties impact social and emotional wellbeing**   - Others reactions and social consequences of coordination - Bullied because of motor coordination issues - Emotional consequences of motor coordination issues - Exclusion - Gender influences experience of coordination issues |
| **Theme 4: Multiple learning and coping strategies are employed**  ***Subtheme: Practice and Learning***   - A persons ability to learn a new motor skill - Practice: The time it takes to learn a new motor skill - Planning and visualisation - Practical strategies   ***Subtheme: Strategies***  ***Subtheme: Avoidance***  ***Subtheme: Experience and need for support.***  [The final 3 subthemes each had one semantic code that was the same name as the subtheme] |
