# Supplementary material for: From “one big clumsy mess” to “a fundamental part of my character.” Autistic adults’ experiences of motor coordination
Source: PLoS One. 2023 Jun 2;18(6):e0286753. doi: 10.1371/journal.pone.0286753 (PMC10237488; doi:10.1371/journal.pone.0286753)
Supplement: S1 Fig — (DOCX) [file pone.0286753.s004.docx]

**S4: Theme development**

**Agreed themes**

1. Motor Coordination difficulties are pervasive and spiky
2. Motor coordination is an active process
3. Motor coordination difficulties impact social and emotional well-being
4. Multiple learning and coping strategies are employed

**Researcher 3 (AW)**

1. The impact of multiple processing on motor coordination
2. Motor Coordination difficulties impact social and emotional wellbeing
3. Motor coordination is pervasive and spiky
4. Strategies used to cope with coordination difficulties

**Researcher 2 (LE)**

1. Coordination difficulties are pervasive and variable.
2. The impact of difficulties on emotional and social wellbeing.
3. Motor coordination is an active process.
4. Strategies created with a lack of support

**Researcher 1 (EG)**

1. Coordination difficulties are pervasive and spiky
2. Variable awareness and understanding
3. Multiple influences on motor coordination
4. Impact on social and emotional well-being
5. Multiple strategies
6. Limited support for motor coordination difficulties
