# Supplementary material for: From “one big clumsy mess” to “a fundamental part of my character.” Autistic adults’ experiences of motor coordination
Source: PLoS One. 2023 Jun 2;18(6):e0286753. doi: 10.1371/journal.pone.0286753 (PMC10237488; doi:10.1371/journal.pone.0286753)
Supplement: Attachment — Submitted filename: Response to reviewers.docx [file pone.0286753.s005.docx]

**1. Please ensure that your manuscript meets PLOS ONE's style requirements, including those for file naming. The PLOS ONE style templates can be found at** 
[**https://journals.plos.org/plosone/s/file?id=wjVg/PLOSOne_formatting_sample_main_body.pdf**](https://journals.plos.org/plosone/s/file?id=wjVg/PLOSOne_formatting_sample_main_body.pdf) **and** 
[**https://journals.plos.org/plosone/s/file?id=ba62/PLOSOne_formatting_sample_title_authors_affiliations.pdf**](https://journals.plos.org/plosone/s/file?id=ba62/PLOSOne_formatting_sample_title_authors_affiliations.pdf)

Confirmed

**2. Thank you for stating the following financial disclosure:**

**Funding was awarded to Emma Gowen through the MRes Experimental Psychology degree programme, The University of Manchester to cover research costs for Louis Earley and Adeeba Waheed. 
 
Please state what role the funders took in the study.  If the funders had no role, please state: "The funders had no role in study design, data collection and analysis, decision to publish, or preparation of the manuscript." Please include this amended Role of Funder statement in your cover letter; we will change the online submission form on your behalf.**

The funders had no role and this has been added to the cover letter.

**a) If there are ethical or legal restrictions on sharing a de-identified data set, please explain them in detail (e.g., data contain potentially sensitive information, data are owned by a third-party organization, etc.) and who has imposed them (e.g., an ethics committee). Please also provide contact information for a data access committee, ethics committee, or other institutional body to which data requests may be sent.**

*There are ethical restrictions that have been included in the cover letter. Consequently, we are unable to share the transcripts. However, the pre-print paper with quotations is currently publicly available (*[*https://psyarxiv.com/76v24/*](https://psyarxiv.com/76v24/)*) and upon publication the paper will also be made publicly available.*

**4. We note that you have stated that you will provide repository information for your data at acceptance. Should your manuscript be accepted for publication, we will hold it until you provide the relevant accession numbers or DOIs necessary to access your data. If you wish to make changes to your Data Availability statement, please describe these changes in your cover letter and we will update your Data Availability statement to reflect the information you provide.**

As noted above, we are unable to share the data (transcripts). However, the paper with quotations will be publicly available. This is explained in the cover letter and the data availability statement has been updated in the paper.

**Reviewer #1:**

**In this study, focus group interviews were used to analyze the performance, existing impact, and corresponding support status of motor coordination disorders in autistic adults. In my view, current study will bring highlights of the problem of motor coordination in autistic adults, and will further pave the way of further understanding of motor coordination related behaviors in autistic adults.**

*Thank you for your supportive comments*

**Even that, several points the author still need to consider.
1. The inclusion criteria of the subjects and the diagnosis of current study should be stated clearly. The current subjects were a sample who could express themselves in words and communicate through the media successfully. To be noticed that that sample is only the minor group of autistic. It will be great to provide the detailed diagnosis information of each subject, and the comorbid (e.g., ADHD, anxiety, depression).**

Inclusion criteria are included and updated on pg 7 under “Participants”. Discussion around the inclusion of additional diagnoses and the sample type is included under limitations in the discussion. We agree that a table listing the details of each participant would be useful, however it would risk making the data identifiable and revealing sensitive information about our participants. For example, it could be possible for people to identify someone from the quotations, then look them up in the table to see what diagnoses they had. As a compromise, we hope that the reviewer will find acceptable that we have indicated in Table 2, which participants score <56 on the ADC using an asterisk.

**2. As the author stated that “A total score equal to or higher than 56 indicates risk of Developmental Coordination Disorder, whereas a score equal to or higher than 65 indicates probable Developmental Coordination Disorder. The average score was 60.3 (32 to 109) with 6 participants below 56, 3 between 56 and 64 and 8 greater than 65.”, there were 6 participants below 56 (minimal was 32) how could the make a conclusion of autistic could experience motor coordination problem? The author should list each subject’s score in a table with what typical motor coordination problem of each include subject having. Otherwise, it will be quite confused how the subjects with low score also show motor coordination problems.**

Thank you for raising this important point. As discussed above it is difficult to provide a table of the diagnoses for each participant, but we have indicated in the table which participants have a score less than 56 so it is clear how participants scoring above or below this cut off described their coordination difficulties. You can see that most of those who scored below 56 still described some difficulties with motor coordination. One exception was P24 who did not describe any motor difficulties.

**3. How did the authors get four themes? The authors should clearly state the concrete process with a flowchart.**

The analysis followed the 6 step technique of Braun and Clarke and is detailed in the analysis section**.** A flow chart has been added to the supplementary materials to describe how the themes from the 3 researchers were narrowed down to 4 final themes.

**4. Whether the number of the participants in the result part can be recoded according to the group order, such as FG1, P1-P5, or whether the specific situation of the participants can be tabulated so that the readers can quickly grasp the specific situation of the specific subjects.**

We have included the participant numbers in table 2

**Reviewer #2: Regarding case selection more than half of them had associated problems like ADHD, anxiety ,depression, Sjogren's syndrome and Ehlers Danlos syndrome. These may have additional affects on motor movements. There was no mention if the movement problems were present from early childhood or developed later or progressively deteriorating. This would have helped to understand the nature of the altered motor coordination.**

Thank you for raising this valid point. As indicated (and expanded upon) in the “participants” section of the methods and “Limitation” section in the discussion we decided to include participants with these additional diagnoses as they are common and we wanted to provide a more representative sample. We agree that it will be important for future work to understand how these might create different/additional motor experiences. In order to show that the motor coordination difficulties in our sample were present from childhood we looked at the first section of the ADC, which contains questions relating to childhood. A score of 17+ indicates past difficulties and approximately half of the participants (8) scored within this range. The remaining ranged between 12 and 16 and when compared to our previous studies, non-autistic people without diagnosed coordination difficulties typically score <10 on this section. Furthermore, for those participants who had an additional diagnoses, only one scored <17 on the childhood section. This shows that motor coordination issues in our group are likely to be from childhood.

These details have been added to the participant section in the methods as well as the limitations*: “However, it is likely that most of the descriptions of motor coordination in our participants relate to long-term difficulties as half scored >17 on the childhood section of the ADC and for those participants who had additional diagnoses, only one scored <17 on the childhood section.”*

**Further the participants status of vision and hearing and sensory profile were not mentioned in the methodology. If the author had taken the above this would have given greater clarity of the association of altered motor coordination with autism.**

This is a good point and one that we would like to follow up in future quantitative work (comparing sensory and motor status). The status of vision and hearing would have been difficult for the current study as it was performed remotely and not in a lab where we could have performed objective testing.

We have included discussion of this in the section called “Relationship with sensory processing”: *“ It would have been informative to collect questionnaire data on sensory experiences of our participants to further understand the links between sensory and motor characteristics and is a direction for future quantitative studies that can collect more detailed and objective sensory and motor data.”*

**There is no data provided of the semantic coding done to comprehend the themes.**

*We have now included a table of the semantic coding in the supplementary materials (Fig S3)*
